# Book Notices and Reviews

**Published:** 1865-06

**Authors:** 


### BOOK NOTICES AND REVIEWS.

*Proceedings of ths American Pharmaceutical Association, at the 12th Annual Meeting, held in Cincinnati, Ohio, Sept.*, 1864. Also, the Revised Constitution and Roll of Members. Merrihew & Son, Printers, Philadelphia. For sale by E. H. Sargent, Manufacturing Pharmaceutist, corner of State and Randolph Streets, Chicago, Ills. Price $1.50.

This is a work of 350 pages; and while it contains much that renders it of the first importance to the Pharmaceutist, it will also be read with profit by the Physician. We have taken the liberty to copy into the present number of the JOURNAL a portion of the Report on Dialysis, by William Proctor, Jr.

---

*The Dispensatory of the United States of America.*—By Geo. B. Wood, M. D., and Franklin Bache, M. D. Twelfth Edition—Carefully Revised. Published by J. B. Lippincott & Co. Philadelphia: 1865. For sale by S. C. Griggs & Co., 39 and 41 Lake Street, Chicago, Ills. Price, $10.

The new edition of the U. S. Dispensatory is at length presented to the profession, by whom, for some time, it has been anxiously looked for. It is issued in the same general style as former editions have been, while above two hundred pages have been added to its matter, and it has been made to conform to the new edition of the U. S. Pharmacopœia, containing all the new official remedies; and such others as, from their general interest, or utility, are thought worthy a place in its pages, and in an abridged form is brought fully up to the present status of the profession in Materia Medica, Therapeutics and Pharmacy. It is a text book in all our Medical Schools, and will be found an essential addition to the library of every practicing Physician.

---

—The American Medical Association will hold its sixteenth annual meeting in Boston, June 16th, 1865.

